# Supplementary material for: How Do Decision Makers and Service Providers Experience Participatory Approaches to Developing and Implementing Physical Activity Interventions with Older Adults? A Thematic Analysis
Source: Int J Environ Res Public Health. 2021 Feb 23;18(4):2172. doi: 10.3390/ijerph18042172 (PMC7926435; doi:10.3390/ijerph18042172)
Supplement: Supplementary file 1 [file ijerph-18-02172-s001.pdf]

Supplementary File S1: Interview Schedule

Greater Manchester Active Ageing Evaluation

Interview Schedule (Providers - Developers)

This interview is to capture your thoughts and experiences on being involved in the Greater Manchester Active Ageing Project. I'll be asking you some general background questions on providing physical activity opportunities for older adults, then I'll focus more on your experiences of the GM Active Ageing project itself. We are interviewing a variety of people in diverse roles so if some of the questions are not pertinent to your experiences, just say so and we will move on. There are no right or wrong answers, I just want to hear your views. You can take a break at any time and you don't have to answer any question you'd rather not. I would ask that you try not to mention other people by name, but if any names are mentioned they will be left out of the transcription. Is that okay?

1. **How long have you been involved in the provision of physical activity to older adults?**
    - What is your professional background?
    - In what role/capacity?
    - What are your responsibilities?

2. **What role did you play in making the decision to take up the Sport England Active Ageing Investment Stream?**
    - What influenced your decision? (if applicable)

3. **What physical activities are you providing/ will you provide for older adults within the Active Ageing Project?**
   Very briefly: where, how, when, with whom are they delivered?
   Different types/ways for different populations? Based on age or ability?
    a. **Why these activities? Did you consider other activities?**
       Are there activities you would like to provide but currently don't? Why? Why not?
    b. **What is it about these activities that you think appeals to older adults?**
       What about physical activities that aren't working out? Why do you think this is?
    c. **How does this differ to previous provision?**

4. **I am going to ask about your experiences of developing physical activity programmes within the Active Ageing Project.**
    - Did you include Co-Design/Co-production/Volunteer peer leaders
    - Did you include Marketing/Acceptability/Behaviour change techniques
    - How did you find using these approaches?
    - What were the challenges? How did you overcome them?
    - What, if anything, facilitated developing the programme? What made it harder?

5. **Why did you make the decisions you did for your Borough Council?**

6. **How did you find the application process for the Active Ageing Project?**
   Were Greater Sport helpful with the application process? In what ways? Was support timely?

7. **At what stage of the project did you receive financial support, e.g. at development stage or later?**
   Why was support received at this stage?
   How did this impact what you offer?

8. **How would you expect context, such as location (particularly thinking of deprived areas) to affect the success of your GM Active Ageing project?**
   Is provision equitable across different demographic areas within your borough?
   How can you address any barriers to equitable provision of physical activity?

9. **What about whether provision is offered directly via the local authority or via an external leisure service or charity? How would you expect that to affect the success of your GM Active Ageing project?**
   What could be the positives/negatives of different approaches?
   What about any impact of the professional background of the providers involved.

10. **Are there any other aspects that you feel will positively or negatively impact the success of the project?**
    Familiar/unfamiliar concepts
    Supportive/unsupportive organisation
    Local culture/local knowledge; Capacity/budgetary issues
    Sport England support/involvement; Greater Sports support/involvement

11. **What would you say is key to successful provision of physical activity for older adults in Greater Manchester?**
    What are key qualities for staff who deliver physical activity to older adults?
    How do you think a service needs to interact with older adults for best results?
    How does the programme fit in with broader context of ageing in Greater Manchester?

12. **Is there anything you would do differently if you had chance to do it again?**
    E.g. When, where, how to deliver and/or market
    More/less: co-design/co-production/training

13. **What would your recommendations be from your experience of developing physical activity projects within the GM Active Ageing Programme?**

14. **What feedback have you received from your staff about the Active Ageing Project? OR How do you think staff are feeling about delivering the project?**

15. **What feedback have you received from older adults about the Active Ageing Project? OR How do you think older adults WILL find the new programme?**

16. **Is there anything else you would like to add?**

Supplementary File S2: Selective, illustrative extract from matrix developed for the working category 'Sustainability'.
Text in square brackets indicates page and line number where issue is discussed (allowing researchers to check where issue arose in the context of the interview, and to facilitate identification of suitable quotations). Underlined text indicates quotations.

**Subcategories/ Interviewees**	**Utilising OAs as assets**	**Continuation of programme when funding ends**	**Cost of Activities**
**P3, MBC Lead**		Would look to sustain the model and how to fund the programme going forward *[p11, 376]* At all times you've got to be thinking about sustainability, what are we going to do when the money's not there from Sport England. *[p14, 470]*	
**P6, MBC Lead**			Cost can be barrier for those living on budget/pension but cost is put on sessions for number of reasons: gives value and people more likely to come back when they know there's a value associated to it, and it helps with the sustainability. Costs help pay for instructor long term and venue hire - without cost it's not sustainable. *[p9, 301]* Cost comes up as barrier through consultations so aim is to keep sessions as low cost as possible to avoid putting people off - do offer initial sessions free but ensure participants aware cost will be introduced *[p13, 450]*
**P7, MBC Lead**	Identifying key individuals in community to upskill to deliver physical activity so there is a legacy left for the programme. Identifying needs of the community gaps and upskilling in the community *[p12, 433]*		Looking at what can be affordable and what can be sustained. The right mix and balance of affordable and free activities. *[p11, 388]*
**P11, MBC Lead**			Found that extra bit of funding is needed to entice people into activity by making it cheap or accessible, which is we can get them into the right programmes which build on social connections, they will stay long term. *[p15, 550]*

**P12, MBC Lead**			First session is free; sessions low cost to encourage people to try activities and stay engaged *[p19, 635]*<br>Activities such as walking groups have no hall hire costs, the activity itself is free *[p21, 695]*
**P15, MBC Lead**		Participants who co-designed the programme concerned about what happens when the funding ends in March 2020. Considering carrying it on by paying small fee or applying for funding. Starting to think about how it will be managed *[p14, 502]*	
**P5, Stakeholder Organisation**	Belief programme will be sustainable because it's things that people actually want to do and they've also co-designed that process and feel a part of something rather than they're just attending as session and leaving, they really feel embedded within the whole system. *[p3, 77]*		
**P8, Stakeholder Organisation**		Challenge for localities after funding ends - is there still a strategic and financial commitment? *[p17, 630]*<br>Would like to think beyond investment period that this way of working is continued but it is a risk and a challenge. Without a Sport England pot of money what does that look like going forward? *[p17, 645]*	Often see funding being used to subsidise activities for set period of time (e.g. £1 sessions jumps up to £6 when funding runs out) - becomes unaffordable to low income participants and straight back into inactivity. Trying to ensure framework emphasises sustainability as key issue. *[p15, 550]*
